# Conjugation of a Blood Brain Barrier Peptide Shuttle to an Fc Domain for Brain Delivery of Therapeutic Biomolecules

**DOI:** 10.1021/acsmedchemlett.1c00225

**Published:** 2021-09-02

**Authors:** Marco Cavaco, Silvia Frutos, Paula Oliete, Javier Valle, David Andreu, Miguel A. R. B. Castanho, Miquel Vila-Perelló, Vera Neves

**Affiliations:** † Instituto de Medicina Molecular João Lobo Antunes, Faculdade de Medicina, Universidade de Lisboa, Av. Prof Egas Moniz, 1649-028 Lisboa, Portugal; ‡ Proteomics and Protein Chemistry Unit, Department of Experimental and Health Sciences, Pompeu Fabra University, Dr. Aiguader 88, Barcelona Biomedical Research Park, 08003 Barcelona, Spain; § SpliceBio S.L., Baldiri Reixac 10-12, 08028 Barcelona, Spain

**Keywords:** Antibody fragments, BBB peptide shuttle, brain disorders, site-specific conjugation, streamlined expressed protein ligation

## Abstract

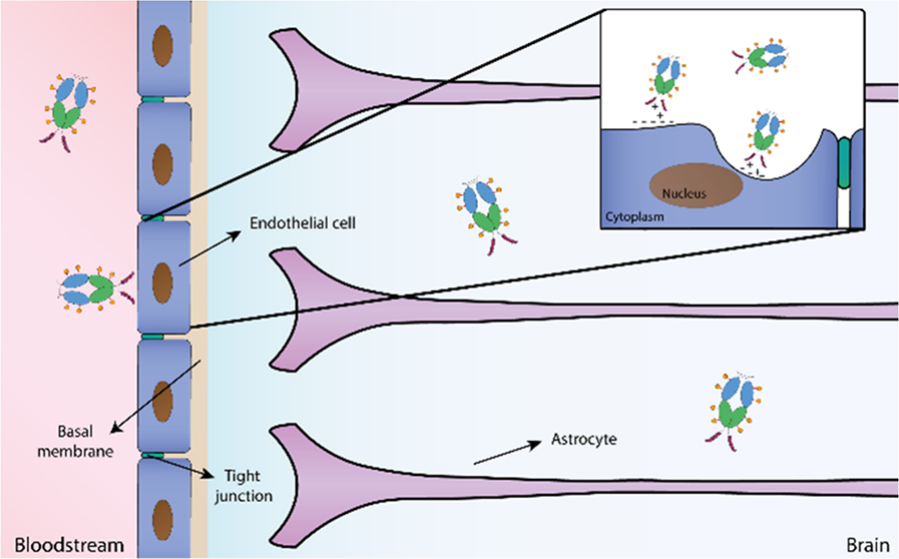

The frequency of brain disease has increased significantly in the past years. After diagnosis, therapeutic options are usually limited, which demands the development of innovative therapeutic strategies. The use of antibody–drug conjugates (ADCs) is promising but highly limited by the existence of the blood–brain barrier (BBB). To overcome the impermeability of this barrier, antibody fragments can be engineered and conjugated to BBB peptide shuttles (BBBpS), which are capable of brain penetration. Herein, we linked the highly efficient BBBpS, PepH3, to the IgG fragment crystallizable (Fc) domain using the streamlined expressed protein ligation (SEPL) method. With this strategy, we obtained an Fc-PepH3 scaffold that can carry different payloads. Fc-PepH3 was shown to be nontoxic, capable of crossing an in vitro cellular BBB model, and able to bind to the neonatal Fc receptor (FcRn), which is responsible for antibody long half-life (*t*_1/2_). Overall, we demonstrated the potential of Fc-PepH3 as a versatile platform readily adaptable to diverse drugs of therapeutic value to treat different brain conditions.

Brain diseases correspond to almost 13% of the global health burden, exceeding cardiovascular and cancer diseases.^1^ Brain cancers (including metastization), stroke, Parkinson’s, and Alzheimer’s are some of the neurological disorders that have increased frequency in the 21st century.^2^ There are some therapeutic protocols that can be administered to these patients to increase their quality of life. However, central nervous system (CNS) therapeutics face a low success rate in disease management, which is somehow related to a general poor knowledge of brain physiology, high frequency of therapeutic adverse events, and absence of certified biomarkers.^3,4^ The existence of the blood–brain barrier (BBB) corresponds to an additional difficulty for effective CNS delivery of numerous small-molecule and biopharmaceutical drugs. The BBB stands as a selective endothelial barrier between the blood and brain compartment that plays a crucial part in brain protection and limits the penetration of therapeutic molecules.^5^

The brain penetration of therapeutic molecules is challenged by barrier properties. In addition, this barrier controls the brain entry of mediators and immune cells. Thus, immune responses within the brain are different compared with other body regions, making the brain an immune privileged site. Hence, treatment of brain conditions remains a concern.^6^

Various innovative strategies to treat different brain conditions have been investigated.^7^ The use of immunotherapy and targeted therapies is the most promising. The former consists on stimulating the host immune system to eliminate nefarious cells,^8,9^ while targeted therapies rely on the binding of an antibody/antibody fragment to an epitope at the membrane of cells or mediators.^10^ The antibody/antibody fragment can be either the therapeutic entity itself, part of a drug-delivery system, such as a nanoparticle or an antibody–drug conjugate (ADCs), or both.

The use of ADCs in cancer treatment has increased in popularity over the years. They display high affinity and tolerability, low drug–drug interactions, and low toxicity.^11^ Other pharmacokinetic (PK) parameters of antibodies, such as high specificity and long half-life (*t*_1/2_), are exploited by drug developers to deliver potent chemotherapeutic agents that by their toxicity or poor PK could not be otherwise administered to patients. Upon conjugation to antibodies/antibody fragments, they show decreasing off-target effects and/or improved PK.^12,13^ Thus, the same strategy can be transposed to other diseases. Unfortunately, CNS-targeted therapies suffer from an important limitation: the brain penetration of drugs, in general, is hampered by the BBB. Thus, without adequate modifications, most therapeutic molecules cannot traverse the BBB and accumulate in the brain, which complicates disease management. Strategies to overcome this limitation and promote BBB antibody crossing include the use of antibody fragments and/or their conjugation to BBB peptide shuttles (BBBpS), which are cell-penetrating peptides (CPPs) that are able to specifically cross endothelial cells and accumulate in the brain.^14^

The initial BBBpS described was the human immunodeficiency virus transactivator of transduction (TAT) peptide,^15^ followed by others, such as SynB, penetratin, Angiopep-2, and dNP2.^16,17^ Our group has contributed with a novel BBBpS, named PepH3, a seven amino acid peptide derived from Dengue virus capsid (DEN2C),^18^ a highly basic protein able to translocate cell membranes.^19,20^ In our in vitro BBB models, PepH3 demonstrated a translocation above 60%,^18,21^ and in vivo studies showed a brain penetration of 0.31% after 5 min injection.^18^ We further evaluated the translocation capabilities of PepH3 upon conjugation to a single domain antibody (sdAb) that recognizes β-amyloid peptide 1–42 (bAP42) (anti-bAP42 sdAb, MW ±14 kDa). After 2 min, a 1.5% (ID/g) brain penetration of the anti-bAP42 sdAb was observed.^22^ The brain uptake values reported are comparable to values obtained for other peptides (<1.1% ID/g)^23,24^ and those of known CNS drugs (e.g., morphine).^25,26^ Moreover, PepH3 demonstrated selectivity toward endothelial cells compared to other cell lines tested (unpublished data), which is a significant advantage since most CPPs are widely distributed upon circulation to different tissues.

Based on these promising data, we decided to pursue PepH3 applications further by conjugating it to a fragment crystallizable (Fc) domain of an immunoglobulin (IgG) (MW ±55 kDa) (Figure 1). This domain is responsible for two of the most important PK/pharmacodynamic (PD) properties attributed to antibodies, namely, long *t*_1/2_, through the neonatal Fc receptor (FcRn) salvage pathway, and increased cytotoxic effect, owing to its capacity to elicit immune effector mechanisms.^27^ Indeed, our Fc-PepH3 platform has attractive PK/PD properties with the capability of penetrating the brain. In addition, the Fc domain provides a scaffold onto which other molecules could be conjugated. The result is a drug-delivery platform that improves the PK properties and efficiency of drugs and that is capable of BBB translocation and brain accumulation (Figure 1).

**Figure 1:**
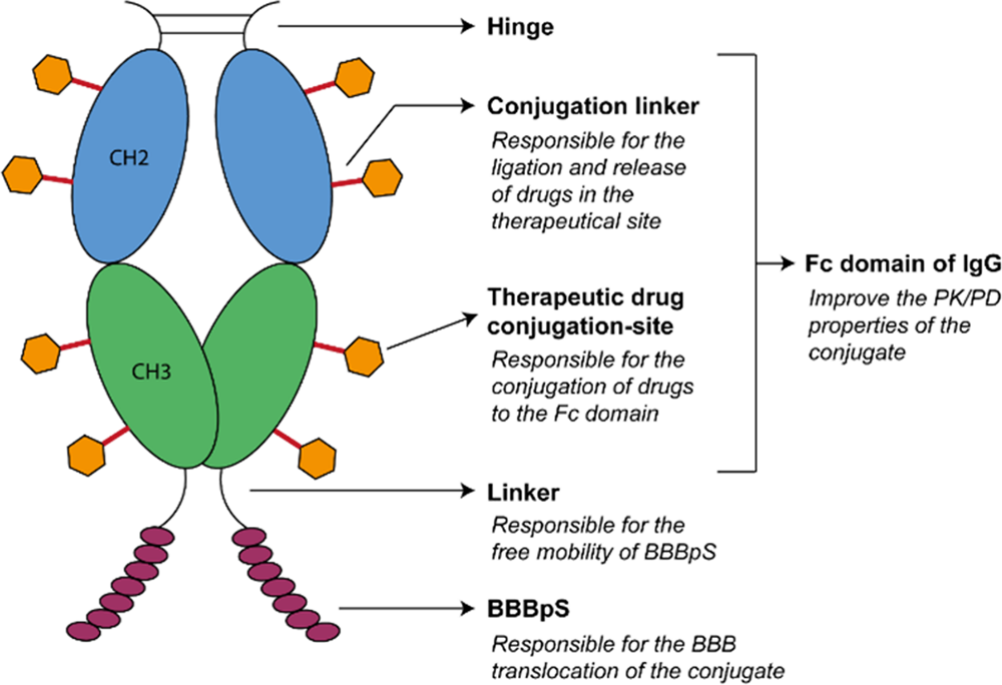
Schematic illustration of the engineered drug platform. Representation of BBBpS conjugated to a generic Fc domain of an IgG1 (MW ±55 kDa). The Fc domain of IgG improves the PK/PD properties of the conjugated drugs, and it has different sites where drugs can be attached via a labile linker that enables their release. The Fc domain is also site-specifically conjugated to a BBBpS, which is responsible for brain penetration. The linker between the peptide and Fc domain allows peptide flexibility, assuring that BBBpS translocation properties are not compromised.

To demonstrate its potential, we addressed the three following main challenges throughout the development of the Fc-PepH3 conjugate: (i) generation of Fc-PepH3 conjugates, (ii) validation of the ability of PepH3 to induce BBB crossing for such a large conjugate, and (iii) ensuring the Fc retains its affinity for the FcRn, responsible for antibody long *t*_1/2_. The first involved the chemical conjugation of the synthetic PepH3 peptide to the Fc domain. Whereas a wide range of chemical methodologies for site-specific conjugation is available,^28−31^ not all of them are ideal for the sensitive nature of peptides and/or Fc, as they tend to rely on experimental conditions potentially detrimental to the species involved (e.g., high temperature/pressure, low/high pH, organic solvents). Thus, we selected the streamlined expressed protein ligation (SEPL), which is run under physiological-like conditions,^32,33^ and yielded a homogeneous conjugate of high purity. A second challenge was to validate the ability of PepH3 to facilitate the in vitro BBB translocation of such a large conjugate. Until now, PepH3 conjugation had involved small payloads.^18,21,22^ Herein, we applied an optimized in vitro BBB model to show the Fc-PepH3 conjugate translocation ability. Third, we wanted to probe the interaction of the Fc domain with FcRn. Altering the binding affinity to this receptor would compromise some of the hoped-for properties mentioned above. In addition, interaction between Fc and FcRn is important for recycling of Fc-PepH3 from the brain to the blood, which results in a get in/get out homeostasis that avoids undesirable toxicity associated with accumulation in the brain.

PepH3 was conjugated to the Fc domain using the SEPL strategy previously described,^34^ a new variant of the expressed protein ligation (EPL) that does not suffer from slow reaction rates and premature hydrolysis.^35,36^ We decided to use SEPL to conjugate the BBBpS PepH3 to the IgG Fc domain (Figure 1), with a goal of using the Fc-PepH3 conjugate as a drug-delivery platform that can be loaded with therapeutic agents, thus favoring drug access to the brain and treatment of brain metastasis.

SEPL requires as building blocks a synthetic thiol-functionalized link, usually a Cys-containing peptide (N-terminal) and a recombinant protein α-thioester. Thus, our first step was N-terminal elongation of the PepH3 sequence with an extra Cys residue and a 6-aminohexanoic acid linker, intended to preserve PepH3 mobility, hence not affecting translocation ability. The solid-phase synthesis was performed by the Fmoc approach in an automated instrument. RP-HPLC/MS confirmed the identity and purity of the peptide (Table S1 and Figure S1). For engineering of the Fc-IntN protein, different ultrafast N-terminal intein fragments might be employed, with Fc-AvaN being the one with the highest rates of Fc-intein fusions and no effects on Fc glycosylation.^34^ To that end, a previously developed plasmid encoding the Fc-AvaN was used, and the recombinant protein was produced in a human expression system (Expi293 cells).

The Fc-peptide conjugation was performed in solution by mixing the Fc-AvaN protein, the IntC fragment, and the Cys-PepH3. The thiolysis/conjugation conditions employed were analogous to those previously optimized.^34^ The success of the reaction was observed by SDS-PAGE and RP-HPLC (Figure 2). To increase the resolution of the samples, they were deglycosylated and reduced to generate monomeric Fc-PepH3 chains before the RP-HPLC analysis. After conjugation, Fc-PepH3 was dialyzed to remove excess reagents and purified by size exclusion chromatography. The Fc-PepH3 conjugate analysis confirmed the existence of an intact Fc dimer (Figure 3A). Moreover, the pure Fc-PepH3 conjugate exhibited the expected MW (Figure 3B). Importantly, no homodimer of the Fc-OH was identified by MS, which proves the value of SEPL.

**Figure 2:**
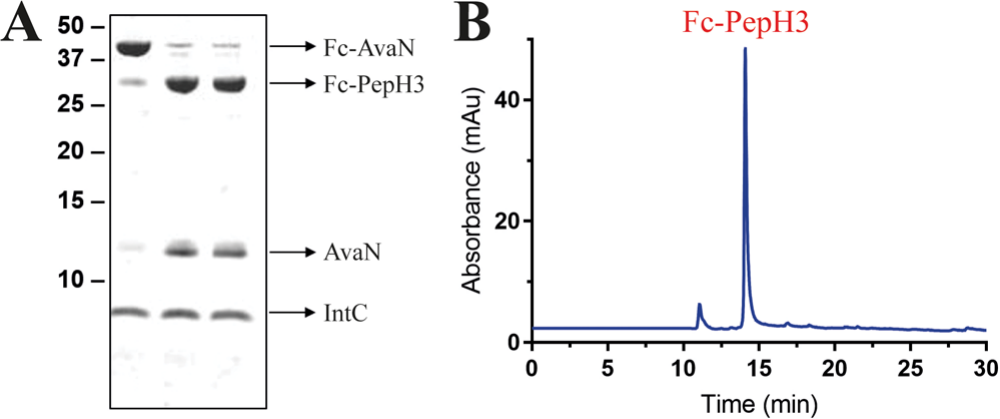
SDS-PAGE and RP-HPLC of Fc-PepH3 reaction mixture. (A) SDS-PAGE analysis of one-spot thiolysis/ligation reaction. Samples of the reaction mixture were taken at 1 min (lane 1) and 24 h (lanes 2 and 3) and analyzed by SDS-PAGE and imaged using Coomassie staining. (B) Thiolysis/ligation reaction analysis by RP-HPLC. Reaction mixture was deglycosylated and fully reduced under denaturing conditions. RP-HPLC analysis of the crude reaction mixture was performed over a 15–40% B gradient on a Zorbax 300SB C8 column.

**Figure 3:**
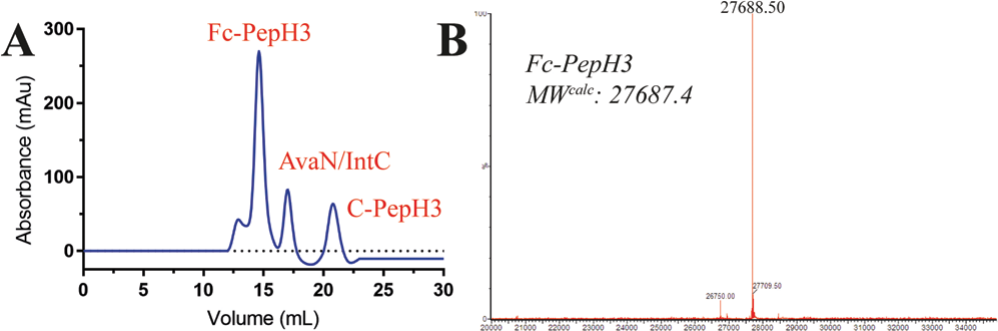
Size-exclusion chromatography (SEC) purification of Fc-PepH3 and LC-MS analysis. (A) SEC purification of thiolysis/ligation reaction mixture over an S200 column at 280 nm. (B) Reaction mixture was deglycosylated and fully reduced under denaturing conditions before the ESI-MS analysis. The presence of a species with a MW in good agreement with the desired, fully reduced Fc-PepH3 was confirmed. No peak corresponding to the hydrolyzed Fc could be detected (MW_calcd_: 26,649.1 Da).

Determining the nontoxic concentration of antibody fragments was important to establish their potential applicability in an in vivo setting. The in vitro toxicity of antibody fragments and conjugates was studied on endothelial (HBEC-5i) and human fibroblast (Hs68) cell lines. We selected HBEC-5i because it is the endothelial cell line used in our in vitro BBB model and human fibroblast Hs68 for its wide application in preclinical toxicity studies.^37^ A CellTiter-Blue assay on both cell lines treated with increasing concentrations for 24 h gave IC_50_ values above 100.0 μM for all antibody fragments (Table 1 and Figure S2). The hemolytic profile was also evaluated using freshly isolated red blood cells (RBCs). This standard safety assay is widely applied due to its simplicity, robustness, cheapness, and highly informative nature.^38^ No hemolytic activity was observed for all antibody fragments (Table 1 and Figure S2).

**Table 1:** Compilation of the Results Obtained with All Antibody Fragments in Different Assays

	hemolytic activity^a^ HC_50_ (μM)	cytotoxicity^b^ IC_50_ (μM)		translocation^c^ (%)		binding affinity^d^*K*_d_(nM)
protein	RBCs	HBEC-5i	Hs68	6 h	24 h	FcRn
Fc-PepH3	>100	>100	>100	17.7 ± 4.34	39.0 ± 7.60	95.4 ± 9.21
Fc-OH	>100	>100	>100	3.6 ± 1.58	16.2 ± 4.53	89.6 ± 8.78
Fc5	>100	>100	>100	4.0 ± 0.56	42.0 ± 3.35	
melittin	0.9 ± 0.12	NA	NA	NA	NA	NA

a Hemolytic activity was determined by absorbance using a plate reader; HC_50_, concentration that causes hemolysis in 50% red blood cells.

b Cellular cytotoxicity was determined using CellTiter-Blue cytotoxicity assay; IC_50_, concentration that causes cell death in 50% of cells.

c Cellular translocation was evaluated by fluorescence intensity using a plate reader.

d Binding affinity was determined by absorbance using a plate reader; *K*_d_, concentration of protein that produces 50% of optimal binding response.

Second, we evaluated the translocation capacity of Fc-PepH3 compared with that of other antibody fragments, which were used as controls. Fc-OH is the unconjugated form of Fc-PepH3, lacking PepH3, which intends to improve BBB translocation, and FC5 was selected based on its well-known translocation properties. In the literature, in vitro BBB models with different levels of complexity are described,^39,40^ and the choice between the different ones depends on the purpose of the study. In our case, we selected a simple and quick in vitro BBB model optimized in our lab.^21,41^ Translocation data obtained with these in vitro BBB models correlate to biodistribution data obtained in vivo.^18^ It consists of two chambers (apical and basolateral) divided by a permeable membrane, which is covered by a monolayer of HBEC-5i cells that in contact with each other creates tight junctions composed of transmembrane proteins.^42^

Data obtained for the Fc-PepH3 conjugate showed it was able to translocate our model, whereas Fc-OH translocation was significantly lower (Table 1, Figure 4, and Figure S3). The 16.2 ± 4.53% Fc-OH found in the basolateral compartment at 24 h might be related to a slightly decreased integrity of the barrier model or to the existence of FcRn receptors at endothelial cells.^43^ In any event, Fc-PepH3 at 6 h displayed translocation 5.0-fold higher than that for Fc-OH, and at 24 h, the percentage of Fc-pepH3 was 2.4-fold higher than that of Fc-OH. Also, the translocation profile of Fc-PepH3 at 24 h was compared with that of recombinant sdAb FC5. FC5 efficiently penetrated the BBB by receptor-mediated transport (RMT),^44^ a mechanism different from the absorptive-mediated transport (AMT) that drives Fc-PepH3 translocation.^18,21^ For FC5, translocation was 4.0 ± 0.56 and 42.0 ± 3.35% at 6 and 24 h, respectively (Table 1 and Figure S3). Furthermore, evaluation of BBB integrity using a 40 kDa dextran conjugated to fluorescein (FD40) revealed that barrier permeability was not compromised by all antibody fragments, excluding a paracellular transport, which validated the data obtained (Figure S2).

**Figure 4:**
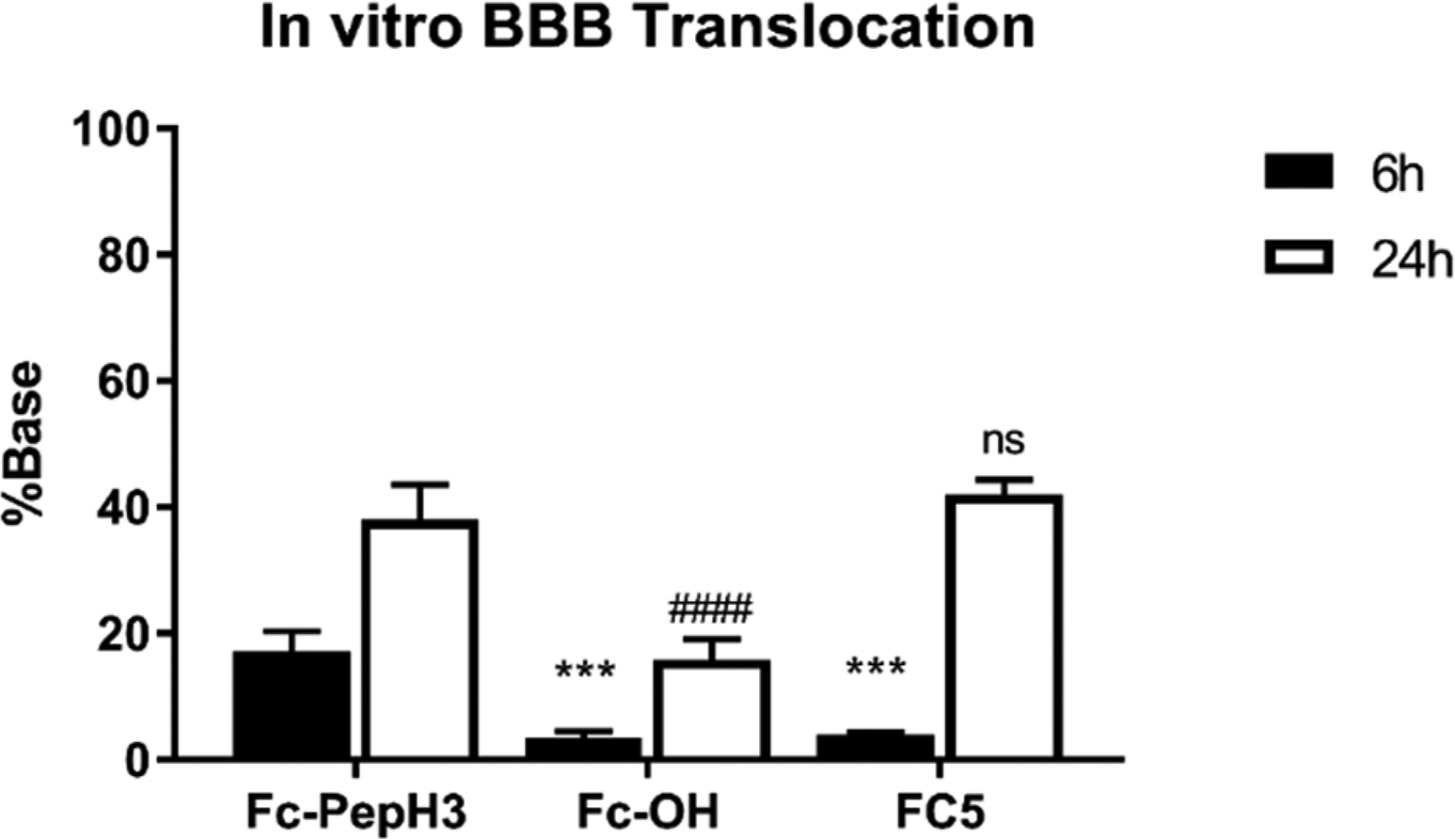
Translocation of antibody fragments across an in vitro BBB model. Percentage of translocation of antibody fragments (25 ng) after 6 and 24 h incubation. The values were obtained from triplicates run in at least three separate days using independently grown cell cultures. Statistical differences were assessed using a two-way ANOVA followed by a Sidak’s multiple test. The “*” is a comparison of samples with Fc-PepH3 after 6h; The “#” is a comparison of samples with Fc-PepH3 after 24 h. Error bars, standard deviation.

The different kinetic profiles observed between Fc-PepH3 and FC5 are likely related to the translocation mechanism. Thus, PepH3 reversibly crosses endothelial membranes by an AMT,^21^ a fast translocation that depends only on the electrostatic interaction between peptide and cellular membranes. In contrast, the RMT of FC5 involves interaction of the antibody fragment with a cell surface receptor. Depending on its distribution, binding affinities, and competition with natural ligands, the translocation occurs at a slower pace and may be irreversible. RMT is still intensely explored,^2,45−47^ with receptor saturation and natural ligand competition as main drawbacks. Therefore, conjugates, such as Fc-PepH3, exploiting alternative BBB translocation pathways such as AMT, are getting increasing attention.^2^ It is also a cornerstone to develop novel therapeutic approaches. In addition, no molecular mass alterations were detected in these assays, which were carried out in serum, demonstrating the stabilities expected for a peptibody.^48^

Finally, in order to assess if conjugation had any effect on Fc *t*_1/2_, the binding affinity of antibody fragments to FcRn was evaluated by ELISA using as control FC5, a sdAb that lacks the Fc domain responsible for interaction with FcRn. As such binding is responsible for the long *t*_1/2_ and cell-mediated toxicity of antibodies, it is essential that no molecule linked to this domain alters the binding ability. Results showed that Fc-PepH3 binds to FcRn at pH 6.0 with a *K*_d_ of 95.4 ± 9.21 nM, comparable to that of Fc-OH (89.6 ± 8.78 nM) (Table 1 and Figure S4). These values are in agreement with previous results,^34^ thus confirming that ligation was functionally successful.

Taken together, our results demonstrate the successful development of a nontoxic Fc-PepH3 platform, able to transverse an in vitro BBB model, using AMT, which is a promising approach that presents some advantages over RMT. Here, we show that (i) the conjugation of a peptide to an IgG Fc domain using a chemical method is a valid approach for in vivo applications; (ii) the Fc-peptide conjugate is capable of effective BBB crossing, to the same extent as a gold standard antibody fragment (FC5), albeit using a different translocation pathway; (iii) the Fc-peptide conjugate keeps the binding affinity of the Fc domain toward the FcRn, which is deemed important to prolonging circulation time and high efficacy; (iv) the Fc-peptide conjugate is nontoxic toward a broad panel of cell lines and erythrocytes, which very likely translates to in vivo safety. Using this conjugate, different therapeutic payloads that could not otherwise be administered due to toxicity can be delivered, improving PK and decreasing off-target effects. The result would be the generation of novel therapeutics with brain penetration ability and retain long *t*_1/2_. The conjugate described here provides a first step toward the development of a novel therapeutic platform to tackle brain disorders to improve patient survival and prognosis.

## Supporting Information Available

Details of the experimental procedures, additional results, and the optimization of the in vitro HBEC-5i model (PDF)
Illustration of Fc-PepH_3_ platform (MP4)

## Abbreviations

ADCs: antibody–drug conjugates
AMT: adsorptive-mediated transport
bAP42: β-amyloid protein-42
BBB: blood–brain barrier
BBBpS: blood–brain barrier peptide shuttle
CNS: central nervous system
CPPs: cell-penetrating peptides
DEN2C: Dengue virus capsid
Fc: fragment crystallizable
FcRn: neonatal Fc receptor
IgG: immunoglobulin
PD: pharmacodynamic
PK: pharmacokinetic
RBCs: red blood cells
RMT: receptor-mediated transport
sdAb: single domain antibody
SEPL: streamlined express protein ligation
TAT: human immunodeficiency virus transactivator of transduction
