# Supplementary material for: Conjugation of a Blood Brain Barrier Peptide Shuttle to an Fc Domain for Brain Delivery of Therapeutic Biomolecules
Source: ACS Med Chem Lett. 2021 Sep 2;12(11):1663–8. doi: 10.1021/acsmedchemlett.1c00225 (PMC9437899; doi:10.1021/acsmedchemlett.1c00225)
Supplement: Supplementary file 1 — ml1c00225_si_001.pdf [file ml1c00225_si_001.pdf]

Conjugation of a Blood Brain Barrier Peptide Shuttle to an Fc domain for brain delivery of therapeutic biomolecules

Marco Cavaco[‡,†], Silvia Frutos[§], Paula Oliete[§], Javier Valle[†], David Andreu[†], Miguel A. R. B. Castanho[‡], Miquel Vila-Perelló[§,*], and Vera Neves[‡,*]

[‡]Instituto de Medicina Molecular João Lobo Antunes, Faculdade de Medicina, Universidade de Lisboa, Av. Prof Egas Moniz, 1649 – 028 Lisboa, Portugal;

[†]Proteomics and Protein Chemistry Unit, Department of Experimental and Health Sciences, Pompeu Fabra University, Dr. Aiguader 88, Barcelona Biomedical Research Park, 08003 Barcelona, Spain;

[§]SpliceBio S.L., Baldiri Reixac 10 – 12, 08028 Barcelona, Spain;

ABSTRACT: The frequency of brain disease has increased significantly in the past years. After diagnosis, therapeutic options are usually limited, which demands the development of innovative therapeutic strategies. The use of antibody-drug conjugates (ADCs) is promising but highly limited by the existence of the blood-brain barrier (BBB). To overcome the impermeability of this barrier, antibody fragments can be engineered and conjugated to BBB peptide shuttles (BBBpS), which are capable of brain penetration. Herein, we linked the highly efficient BBBpS, PepH3, to the IgG fragment crystallizable (Fc) domain using streamlined expressed protein ligation (SEPL) method. With this strategy, we obtained an Fc-PepH3 scaffold that can carry different payloads. Fc-PepH3 was shown to be non-toxic, capable of crossing an *in vitro* cellular BBB model, and binding to neonatal Fc receptor (FcRn), which is responsible for antibodies long half-life ($t_{1/2}$). Overall, we demonstrated the potential of Fc-PepH3 as a versatile platform readily adaptable to diverse drugs of therapeutic value to treat different brain conditions.

KEYWORDS: *Antibody fragments; BBB peptide shuttle; brain disorders; site-specific conjugation; streamlined expressed protein ligation*

Table of Content

1. Experimental Procedures S4

a. Materials S4

b. Peptide synthesis and purification S4

Figure S1 | RP-HPLC and MS analysis of the purified Cys-PepH3. (**A**) RP-HPLC of purified Cys-PepH3 over a 10 – 50% B gradient on a Luca C18 column (220 nm detection). (**B**) MS of the entire HPLC peak. The presence of a species with a MW in good agreement with the Cys-PepH3 peptide. No peaks corresponding to impurities were detected (MW_{Calc}: 1,059.3 Da). S5

Table S1. Peptides synthetized. S6

c. Expression and purification of Fc-IntN S6

d. Thiolysis/ligation of Cys-PepH3 to the Fc-IntN S6

e. Characterization of Fc-PepH3 conjugate S6

f. Hemolytic activity S7

g. Cell culture S7

h. Toxicity towards human cell lines S7

i. Translocation across a human endothelial cell line S8

j. In vitro BBB model integrity assay S8

k. FcRn binding affinity S9

l. Statistical analysis S9

2. Results S10

Figure S2. *in vitro* toxicity of antibody fragments. HBEC-5i, Hs68, and RBCs were incubated with 100 µL of previously diluted antibody fragments (0.001 – 100.0 µM range) for 24h. The values were obtained from triplicates of three independent experiments. Error bars, S.D. S10

Figure S3. Translocation of antibody fragments across an *in vitro* BBB model and the FD40 permeability study. Percentage of translocation of antibody fragments (25 ng) and fluorescence intensity of FD40 measured after translocation assay. The values were obtained from triplicates of three independent experiments. Error bars, S.D. S11

Figure S4. Binding of proteins to the FcRn. Antibody fragments' binding curves to immobilized FcRn at pH 6.0, obtained using a single-site fit model. The values were obtained from triplicates of three different experiments. Error bars, S.D. S12

3. Supplementary data – *in vitro* HBEC-5i characterization S13

Figure S5. Confocal images of tight junction proteins in HBEC-5i cells. (**A**) Control, (**B**) Claudin-5, (**C**) Occludin-1, and (**D**) ZO-1. Images were obtained using a 40x water-objective. Nuclei stained with Hoeschst 33342 (blue) and tight junction proteins (red). Bar represents 10 µm. 13

Figure S6. Transport of Lucifer Yellow (LY) across HBEC-5i monolayers. Ly applied at a concentration of 50.0 µM in serum-free medium. HBEC-5i monolayers were incubated with LY in or without the presence of EGTA. Experiments were performed in triplicates on different days using independently grown cell cultures. Data is shown as mean±SD. S14

Figure S7. Transport of human Transferrin (hTf) across HBEC-5i monolayers. hTf applied at a concentration of 400.0 µg/mL in serum-free medium. HBEC-5i monolayers were incubated with hTf (24h) in or without the presence of EGTA. Experiments were performed in triplicates on different days using independently grown cell cultures. Data is shown as mean±SD S15

Figure S8. Confocal images of HBEC-5i cells incubated for 20 min at 37°C with Human Transferrin at a concentration of 25 µg/mL in HEPES supplemented with 1 M glucose, 0.2 M $MgCl_2$, and 1% BSA. (**A – B**) 20x air-objective, (**C – D**) 63x oil-objective. Nuclei stained with Hoeschst 33342 (blue) and transferrin Alexa Fluor 568 conjugate (red). Bar represents 10 µm. S16

SUPPORTING INFORMATION

1. Experimental Procedures

a. Materials

Fmoc-protected amino acids, Fmoc-Rink amide (MBHA) resin, 2-(1H-benzotriazol-1-yl)-1, 1,3,3-tetramethyluronium hexafluorophosphate (HBTU), and N-hydroxybenzotriazole (HOBt) were from Iris Biotech (Marktredwitz, Germany). HPLC-grade acetonitrile (ACN), and peptide-synthesis grade N,N-dimethylformamide (DMF), dichloromethane (DCM), N,N-diisopropylethylamine (DIEA), N,N-diisopropylcarbodiimide (DIPCI), trifluoroacetic acid (TFA), and triisopropylsilane (TIS) were from Carlo Erba-SDS (Sa-badell, Spain). 3,6-dioxa-1,8-octanedithiol (DODT), and fluorescein isothiocyanate-40 KDa dextran (FD40) were from Sigma-Aldrich (Madrid, Spain).

Opti-minimal essential medium (Opti-MEM®), Dulbecco's Modified Eagle Medium (DMEM), DMEM/Ham's F-12 (DMEM:F12), DMEM:F12 without phenol-red, trypsin-EDTA, attachment factor protein solution (AF), fetal bovine serum (FBS), penicillin-streptomycin antibiotic solution (Pen/Strep), and Expi293 Expression System were from Gibco/Thermo Fischer (Gaithersburg, Maryland, USA). Endothelial cell growth supplement (ECGS) was from Sigma-Aldrich (Madrid, Spain). CellTiter-Blue® Cell Viability Reagent was from Promega (Madrid, Spain).

All buffering salts, isopropyl-β-D-thiogalactopyranoside (IPTG), Tris(2-carboxyethyl)phosphine hydrochloride (TCEP), and N,N-diisopropylethylamine (DIPEA) were from Thermo Fisher (Gaithersburg, Maryland, USA). Sodium 2-mercaptoethanesulfonate (MES), ethanedithiol (EDT), and Coomassie brilliant blue were from Sigma-Aldrich (Madrid, Spain). Complete protease inhibitor tables were from Roche Diagnostics (Mannheim, Germany). Criterion XT Bis-Tris gels (12%), Immuno-blot PVDF membrane (0.2 µm), Immuno-blot nitrocellulose membrane, Bradford reagent dye concentrate, Tris/Glycine/SDS running buffer, Tris/Glycine transfer buffer were from Bio-Rad (Hercules, California, USA). ECL Prime western blotting detection reagent from GE Healthcare (Chi-cago, Illinois, USA). Goat anti-human IgG-HRP from Abcam (Cambridge, UK).

b. Peptide synthesis and purification

Cys-PepH3 (C(Ahx)AGILKRW-amide) was synthetized in a Prelude Synthesizer (Gyros Protein Technologies, USA) running Fmoc (FastMoc) SPPS protocols at 0.1 mmol scale on a Fmoc-Rink-amide ChemMatrix resin (**Table S1**). Side chain functionalities were protected with NG-2,2,4,6,7-pentamethyldihydrobenzofuran-5-sulfonyl (Arg), Nα-tert-butoxycarbonyl (Trp), and trityl (Cys) groups. Eight-fold excess of Fmoc-L-amino acids and HBTU, in the presence of a double molar amount of DIEA, were used for the coupling steps, with DMF as solvent. After chain assembly, full depro-tection and cleavage were carried out with TFA/H2O/DODT/TIS (94:2.5:2.5:1, v/v, 90 min, rt). Peptides were isolated by precipitation with cold diethyl ether and centrifugation at 4,000 xg, 4°C for 20 min. Then, they were taken up in H2O and lyophilized.

Analytical reversed-phase (RP) HPLC was performed on a Luna C18 column (4.6 x 50 mm, 3 µm; Phenomenex, Tor-rance, California, USA) using linear gradients of solvent B (0.036% TFA in MeCN) into solvent A (0.045% TFA in H2O) at a flow rate of 1 mL/min and with UV detection at 220 nm. Preparative RP-HPLC was performed on a Luna C18 column (21.2 x 250 mm, 10 µm; Phenomenex, Torrance, Cali-fornia, USA) using linear gradients of solvent B (0.1% TFA in MeCN) into solvent A

(0.1% TFA in H2O) with a flow rate of 25 mL/min and with UV detection at 220 nm (**Figure S1A**). LC-MS was performed in a LC-MS 2010EV instru-ment (Shimadzu, Kyoto, Japan) fitted with an XBridge C18 column (4.6 x 150 mm, 3.5 µm; Waters, Spain), eluting with linear gradients of HCOOH/MeCN (0.08% v/v) into HCOOH/H2O (0.1% v/v) over 15 min at 1 mL/min (**Figure S1B**). Peptide stock solutions (1 mM) in filtered H2O were stored at -20°C.

A

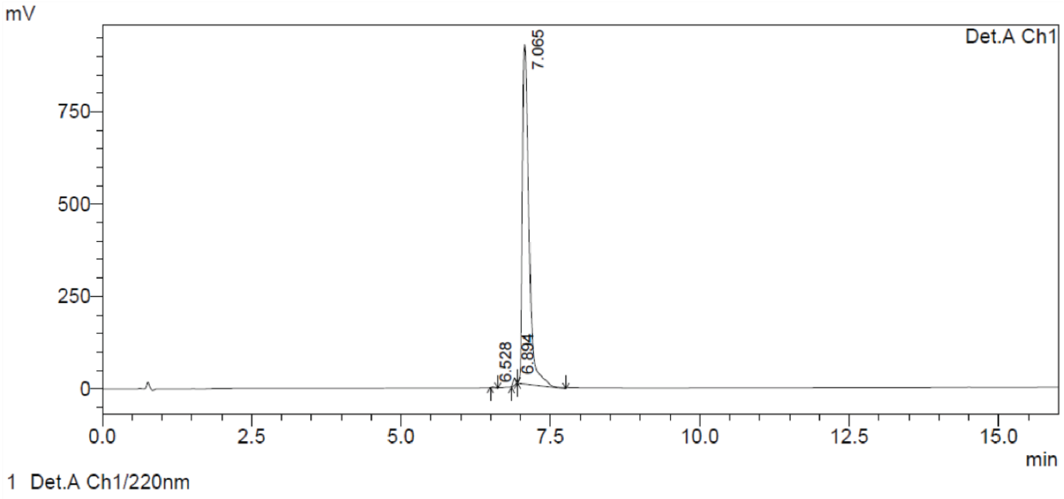

PeakTable

Detector A Ch1 220nm

Peak#	Ret. Time	Area	Height	Area %	Height %
1	6.528	15958	3051	0.230	0.324
2	6.894	64531	18859	0.929	2.005
3	7.065	6868959	918652	98.842	97.671
Total		6949448	940562	100.000	100.000

B

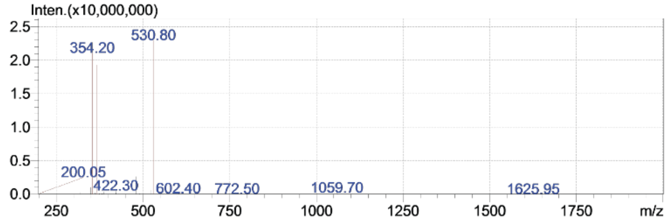

Figure S1 | RP-HPLC and MS analysis of the purified Cys-PepH3. (A) RP-HPLC of purified Cys-PepH3 over a 10 – 50% B gradient on a Luca C18 column (220 nm detection). **(B)** MS of the entire HPLC peak. The presence of a species with a MW in good agreement with the Cys-PepH3 peptide. No peaks corresponding to impurities were detected (MW_{Calc}: 1,059.3 Da).

Table S1. Peptides synthetized.

Peptide	Amino Acid Sequence	Theoretical mass (Da)[a]	Experimental mass (Da)[b]	HPLC t_R (min)	Purity (%)[c]
C-PepH3	C(Ahx)AGILKRW-amide	1059.3	1060.6	7.065	97.7

[a]Calculated using GPMAW version 8.10
[b]Determined from the MS spectra
[c]Estimated by peak integration of the analytical HPLC chromatograms
Ahx, aminohexanoic acid

c. Expression and purification of Fc-IntN

Expi293 cells were transiently transfected with Fc-IntN constructs using Expi293 Expression System, according to the manufacturer's instructions. Briefly, cells were grown in suspension in Expi293 Expression Medium, in vented-cap shaker flasks for 5 days at 37°C. Prior to the day of transfection, Expi293 cells were seeded at 2.5 x 106 cells/mL. The day of transfection, Expi293 cells were seeded at 3.0 x 10^6 cells/mL. Transfection complexes were prepared by mixing 80 µl of ExpiFectamine 293 transfection reagent with 30 µg of plas-mid DNA in 1.5 mL Opti-MEM® medium. After 20 min incubation at r.t., transfection mixtures were added to a suspension of Expi293 cells in 30 mL volume. After 24h, 150 µL of ExpiFectamine transfection Enhancer 1 and 1.5 mL of ExpiFectamine Transfection Enhancer 2 were added to the transfected cells.

After 5 days incubation, cell supernatant was harvested and spun down at 2,000 xg for 20 min at 4°C and filtered through a 0.22 mm filter to remove any cell debris. After addition of Complete Protease Inhibitors, cell supernatant was directly purified over the Ni-beads using the standard protocol, after which the eluted Fc-IntN was dialyzed into 1x PBS (137 mM NaCl, 2.7 mM KCl, 10 mM Na2HPO4, and 1.8 mM KH2PO4, pH 7.4) and 1 mM EDTA.

d. Thiolysis/ligation of Cys-PepH3 to the Fc-IntN

Ni-beads purified Fc-IntN at 500 µg/mL was mixed with 25 µM of IntC and 0.5 mM Cys-PepH3 in 100 mM phosphate, 150 mM NaCl, 1 mM EDTA, 100 mM MESNa, 1 mM TCEP, pH 7.2. Reaction was allowed to proceed, protected from light at r.t. for 24h. The reaction endpoint was monitored by SDS-PAGE, then the reaction mixture was extensively dialyzed into 1x PBS to remove excess peptide and MESNa. The Fc-PepH3 conjugate was then purified over a Superdex S200 column on an Akta purification system (GE Healthcare; Chicago, Illinois, USA).

e. Characterization of Fc-PepH3 conjugate

Fc-PepH3 conjugate was analyzed by RP-HPLC/MS and size-exclusion chromatography. 50 µg of Fc-PepH3 was used for RP-HPLC/MS analysis. Before the analysis, the sample was deglycosylated and fully reduced. Deglycosylation was performed using 2U of PNGase F per µg of sample and incu-bated at 37 °C o.n, then denatured by exchanging sample buffer to 6M Gn-HCl, 100 mM phosphate, 150 mM NaCl, 1 mM EDTA at pH 7.2 and fully reduced by treatment with 10 mM DTT at 37°C for 1h. The analysis was performed on a Zorbax 300SB C8 column using a 15-70% linear gradient of solvent B into solvent A over 30 min at 1 mL/min flow rate and 70°C, preceded by 3 min isocratic elution at

15% B. Sol-vent A was 0.02% TFA and 0.25% FA (formic acid) in H2O. Solvent B was 90% isopropanol with 0.02% TFA and 0.25% FA in H2O. HPLC peaks were collected and analyzed by MS.

The integrity of Fc-PepH3 conjugate was confirmed by in-jecting 50 µg of ligated and dialyzed conjugate in running buffer (100 mM phosphate, 150 mM NaCl, 1 mM EDTA, 1 mM TCEP, pH 7.2) into an S200 size-exclusion column in the Akta purification system.

f. Hemolytic activity

Fresh human blood was collected in EDTA tubes and centrifuged at 1,000 xg for 10 min at 4°C. The supernatant was discharged, and the pellet containing RBCs was washed three times with 1x PBS and resuspended in 1x PBS to obtain a 2.0% (v/v) suspension. Then, RBCs were added to centrifuge tubes containing 2-fold serially diluted conjugates to a final concentration ranging from 0.001 to 100 µM. The suspension was incubated for 24 h at 37°C with gentle stirring. After that, samples were centrifuged for 2 min at 1,000 xg. Supernatants were transferred to 96-well plates, and the hemoglobin released measured by absorbance at 570 nm in a Varioskan™ LUX multimode microplate reader (Thermo Fisher, Madrid, Spain). 1x PBS with no peptides and Triton X-100 at 1% and 4% (v/v) were used as negative and positive controls, respectively. Hemolytic activity (%) was determined using the following equation:

$$Hemolysis\ (\%) = \left(\frac{Abs_{PT} - Abs_{NC}}{Abs_{PC} - Abs_{NC}}\right) \times 100 \qquad \text{Eq. 1}$$

Abs_{PT} is the absorbance of treated samples, Abs_{NC} is the absorbance from negative control, and Abs_{PC} absorbance from positive control.

HC_{50} values were determined using the GraphPad Prism 7.0 software using a log(inhibitor) vs. normalized response. Experiments were performed on different days using independent blood donors.

g. Cell culture

Adherent human brain endothelial cells (HBEC-5i, ATCC® CRL-3345TM) were grown in AF-coated T-flasks as monolayer in DMEM:F12 supplemented with 10% FBS, 1% pen-strep, and 15 µg/mL ECGS. Adherent human fibroblasts cells (Hs68, ATCC® CRL-1635TM) were grown as monolayer in DMEM supplemented with 10% FBS, and 1% pen-strep. Cells were cultured in 95% humidified atmosphere and 5% CO2 at 37 °C (MCO-19AIC (UV), Sanyo, Japan), with the medium changed every other day.

h. Toxicity towards human cell lines

Protein's cytotoxicity towards HBEC-5i and Hs68 cell lines was determined using the CellTiter-Blue® cell viability assay, following a described protocol. Briefly, HBEC-5i and Hs68 were carefully harvested with trypsin-EDTA and seeded at 15,000 cells/100 µL into 96-well clear flat-bottomed polystyrene plates (Corning, New York, USA) for 24h. After medium removal, cells were washed two

times with 1x PBS, and 100 µL of antibody fragments (0.001 – 100 µM range) in the respective medium were added to cells. After 24h, cells were washed two times with 1x PBS and 20 µL of CellTiter-Blue® reagent (diluted in 100 µL medium) was added to each well and incubated for 3h in culturing conditions. The fluorescence intensity was measured using VarioskanTM LUX multimode microplate reader (Thermo Fisher, Madrid, Spain).

IC_{50} values were determined using GraphPad Prism 7.0 software using a log(inhibitor) versus normalized response. Experiments were performed in triplicates on different days using independently grown cell cultures.

i. Translocation across a human endothelial cell line

The in vitro translocation capacity of antibody fragments was evaluated using an in vitro HBEC-5i cell model, as previously described. Briefly, HBEC-5i cells were carefully harvested with trypsin-EDTA and seeded 8000 cells/well in pre-coated tissue culture inserts (transparent polyester (PET) membrane with 1.0 µm pores) for 24-well plates (BD Falcon, New York, USA). During 8 days, medium was changed every other day. After medium removal, cells were washed two times with 1x PBS and one time with DMEM:F12 medium without phenol red. Then, 25 ng of previously diluted anti-body fragments were added to the apical side of the in vitro BBB model and incubated for 6 and 24h. Then, samples were collected from the apical and basolateral side and loaded onto 12% acrylamide Bis-Tris gels and run in Tris/Glycine/SDS running buffer (25 mM Tris, 192 mM Glycine, 0.1% SDS, pH 8.3). The resolved antibody fragments were transferred from the gel onto a nitrocellulose membrane in Tris/Glycine transfer buffer (25 mM Tris, 192 mM Glycine, 20% (v/v) methanol, pH 8.5) at 250 mA for 90 min. Membranes were blocked with 5% low fat milk in 1x PBST, then blotted with a HRP-preadsorbed anti-human IgG antibody (1:10,000, in 1x PBS). The blots were revealed using ECL Prime Western Blotting Detection Reagent according to the manufacturer´s instructions. Then, imaged using the Amersham Imager 600 (GE Healthcare; Chicago, Illinois, USA). Experiments were performed on different days using independently grown cell cultures.

j. In vitro BBB model integrity assay

In the end of the translocation assay, an in vitro BBB integrity assay was performed. Herein, cells were washed two times with 1x PBS and one time with DMEM:F12 medium without phenol red. Then, previously diluted FD40 was added to the apical side and incubated for 2h. FD40 was diluted in DMEM:F12 medium without phenol red to an absorbance below 0.1. Finally, samples from the apical and basolateral side were collected and fluorescence intensity analyzed using a Varioskan™ LUX multimode microplate reader. The percentage of FD40 recovered was determined using the following equation:

$$FD40\ Permeability\ (\%) = \left(\frac{F_i - F_{cells}}{F_{FD40} - F_{Medium}}\right) \times 100 \qquad \text{Eq. 2}$$

F_i is the fluorescence intensity recovered, F_{cells} is the fluorescence intensity recovered from cells without treatment, F_{FD40} is the fluorescence intensity of total FD40 initially added to the transwell apical side, and F_{Medium} is the fluorescence intensity of the medium.

The integrity of the in vitro BBB model is indirectly proportional to the percentage of FD40 recovered and was determined using the following equation:

$$Integrity(\%) = 100 - FD40\ Permeability\ (\%) \qquad \text{Eq. 3}$$

k. FcRn binding affinity

96-well polystyrene ELISA plates (Corning, USA) were coated with 2 µg/mL (100 µL/well) FcRn (R&D Systems; Minneapolis, USA) diluted in 100 mM NaPO4, pH 6.0, 0.05% Tween 20 (v/v), and 0.1% BSA (m/v) at 4ºC o.n. The coated wells were then incubated with a blocking solution (0.5% BSA) for 2h at r.t., washed 4 times and incubated for 1h with serially diluted antibody fragments at r.t. (0.1 – 10,000 nM range). After 4 more washes, HRP-conjugated goat anti-polyhistidine antibody (1:10,000) was added to the plates for protein detection. After another 4 washed, the plate was developed by adding 50 µL of Ultra-TMB substrate to each well. The absorbance was measured at 450 nm using a VarioskanTM LUX multimode plate reader. The binding data were GraphPad Prism 7.0 software using a single-site kinetic fit model. Experiments were performed in triplicates.

l. Statistical analysis

Quantitative data were processed using Excel 2013 (Mi-crosoft; New York, USA) and the GraphPad Prism 7.0 soft-ware package. Medians, means and standard deviations are shown in the figures and tables. Pairwise significances were calculated using one-way ANOVA followed by Tukey's multiple comparison test, nonparametric Mann–Whitney, Kruskal–Wallis, and two-way ANOVA by Sidak's multiple comparison test.

2. **Results**

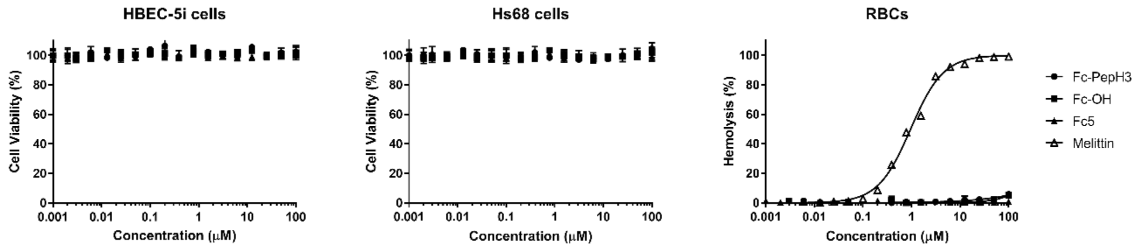

Figure S2. *in vitro* toxicity of antibody fragments. HBEC-5i, Hs68, and RBCs were incubated with 100 µL of previously diluted antibody fragments (0.001 – 100.0 µM range) for 24h. The values were obtained from triplicates of three independent experiments. Error bars, S.D.

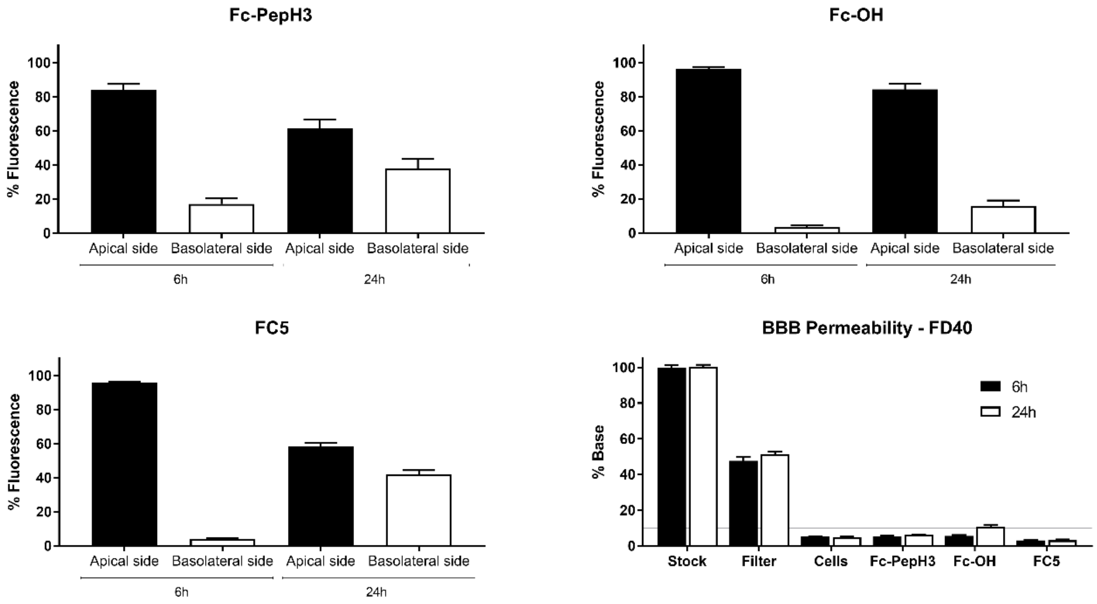

Figure S3. Translocation of antibody fragments across an *in vitro* BBB model and the FD40 permeability study. Percentage of translocation of antibody fragments (25 ng) and fluorescence intensity of FD40 measured after translocation assay. The values were obtained from triplicates of three independent experiments. Error bars, S.D.

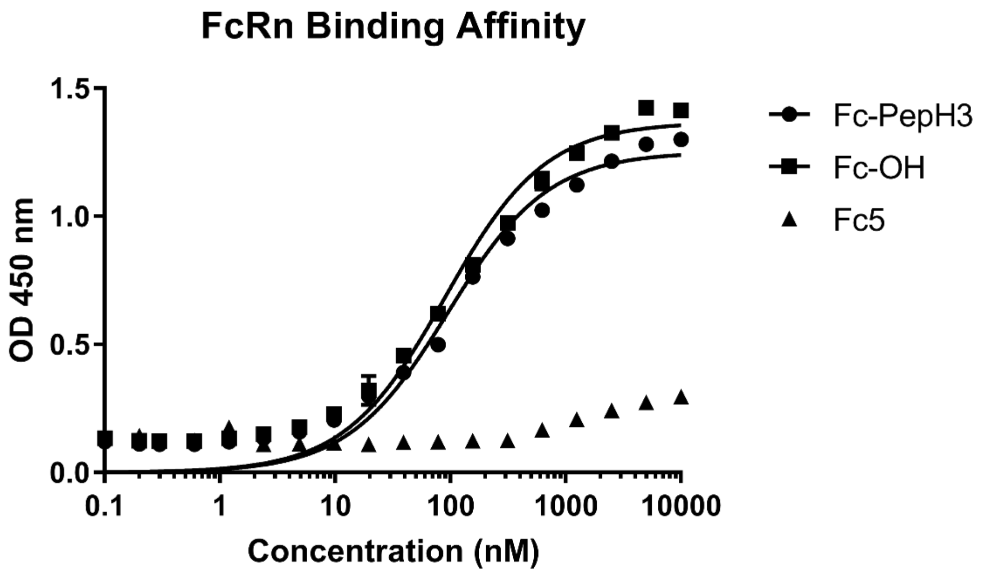

Figure S4. Binding of proteins to the FcRn. Antibody fragments' binding curves to immobilized FcRn at pH 6.0, obtained using a single-site fit model. The values were obtained from triplicates of three different experiments. Error bars, S.D.

3. Supplementary data – *in vitro* HBEC-5i characterization

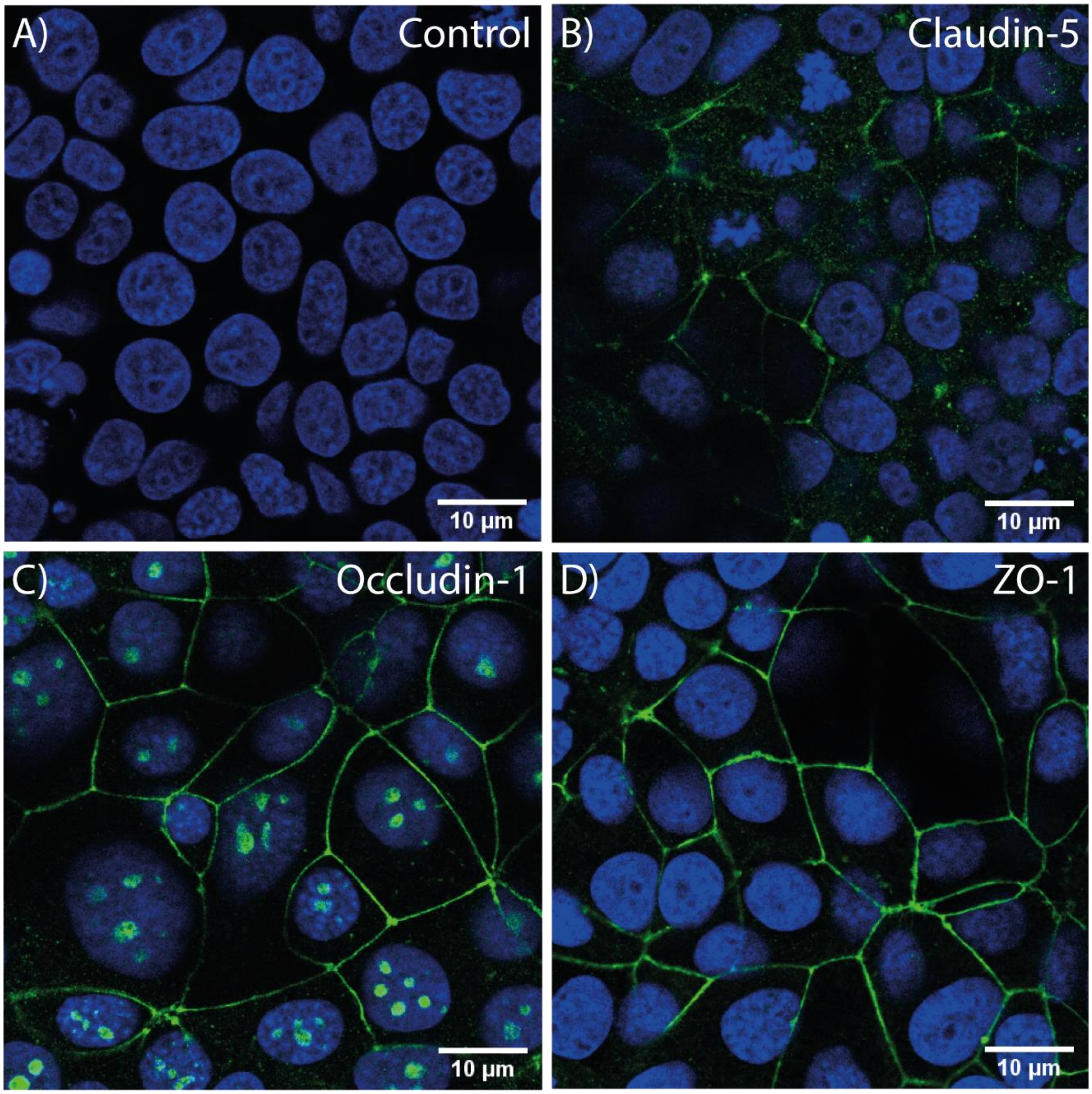

Figure S5. Confocal images of tight junction proteins in HBEC-5i cells. (A) Control, **(B)** Claudin-5, **(C)** Occludin-1, and **(D)** ZO-1. Images were obtained using a 40x water-objective. Nuclei stained with Hoeschst 33342 (blue) and tight junction proteins (red). Bar represents 10 µm.

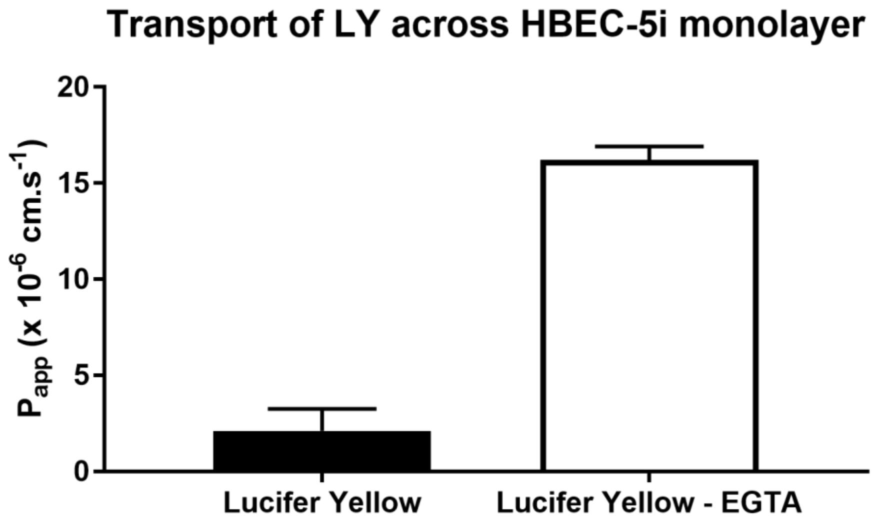

Figure S6. Transport of Lucifer Yellow (LY) across HBEC-5i monolayers. Ly applied at a concentration of 50.0 µM in serum-free medium. HBEC-5i monolayers were incubated with LY in or without the presence of EGTA. Experiments were performed in triplicates on different days using independently grown cell cultures. Data is shown as mean±SD.

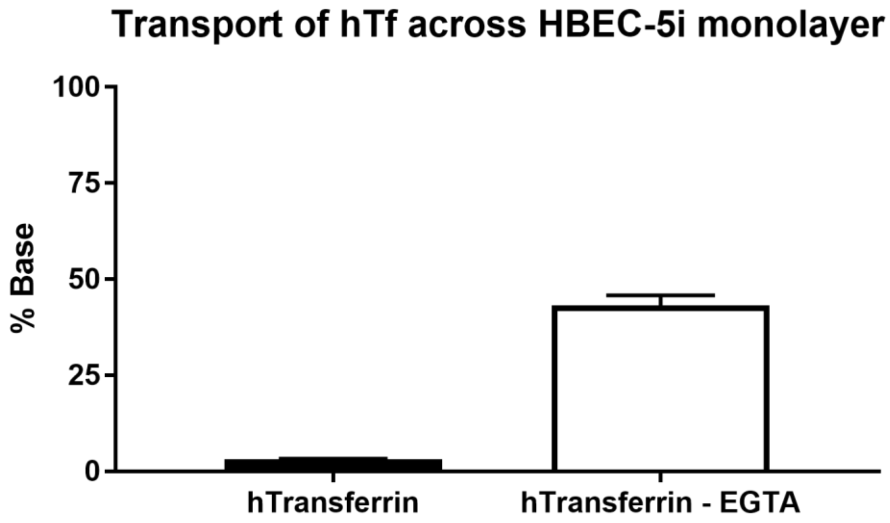

Figure S7. Transport of human Transferrin (hTf) across HBEC-5i monolayers. hTf applied at a concentration of 400.0 µg/mL in serum-free medium. HBEC-5i monolayers were incubated with hTf (24h) in or without the presence of EGTA. Experiments were performed in triplicates on different days using independently grown cell cultures. Data is shown as mean±SD

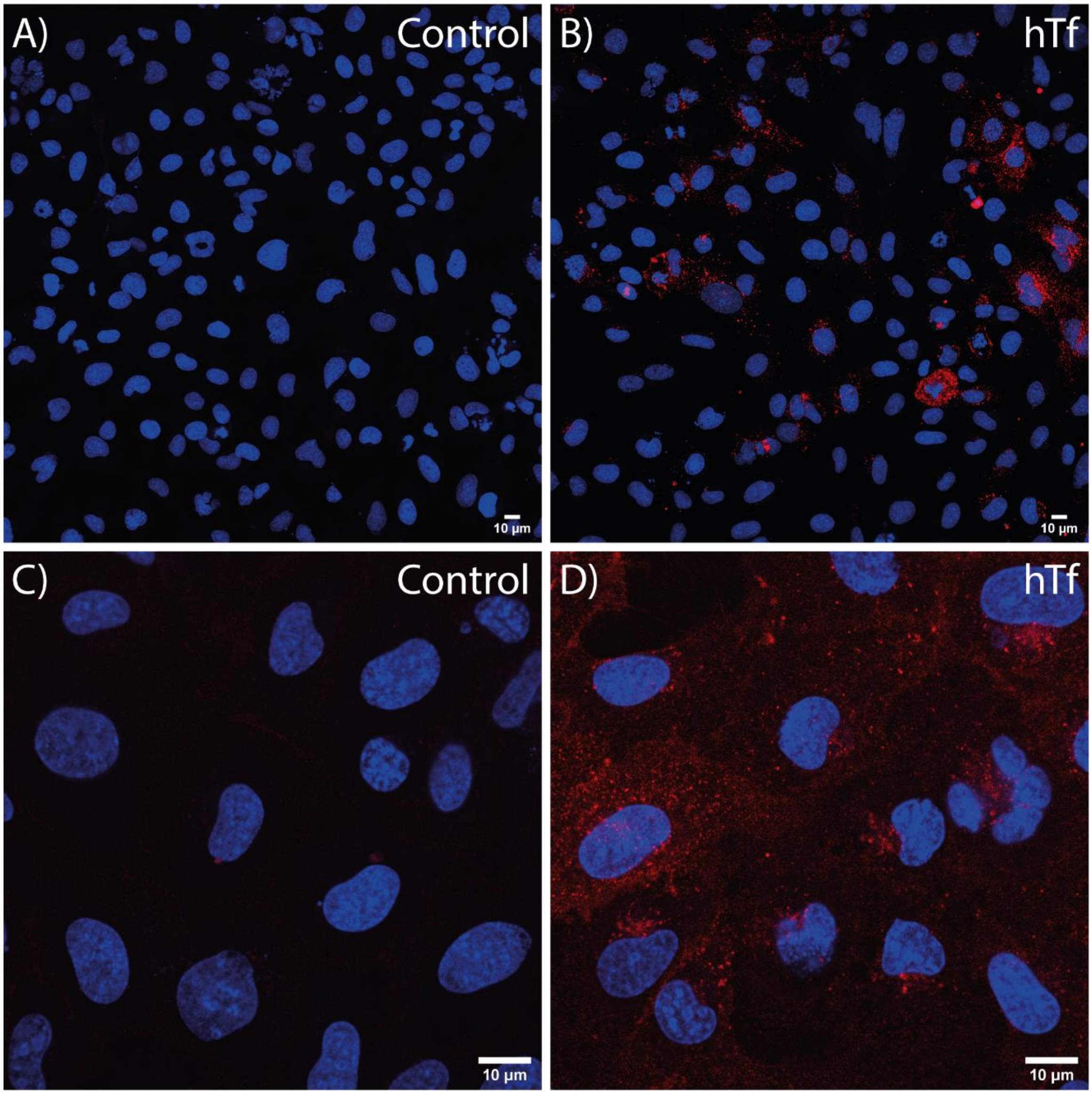

Figure S8. Confocal images of HBEC-5i cells incubated for 20 min at 37°C with Human Transferrin at a concentration of 25 µg/mL in HEPES supplemented with 1 M glucose, 0.2 M $MgCl_2$, and 1% BSA. (A – B) 20x air-objective, **(C – D)** 63x oil-objective. Nuclei stained with Hoeschst 33342 (blue) and transferrin Alexa Fluor 568 conjugate (red). Bar represents 10 µm.
